# B‐prolymphocytic leukemia (B‐PLL) with genetic aberrations involving *MYC* and *TP53*

**DOI:** 10.1002/jha2.298

**Published:** 2021-10-03

**Authors:** Gurdip Singh Tamber, Ramya Gadde

**Affiliations:** ^1^ Case Western Reserve University/University Hospitals Cleveland Medical Center


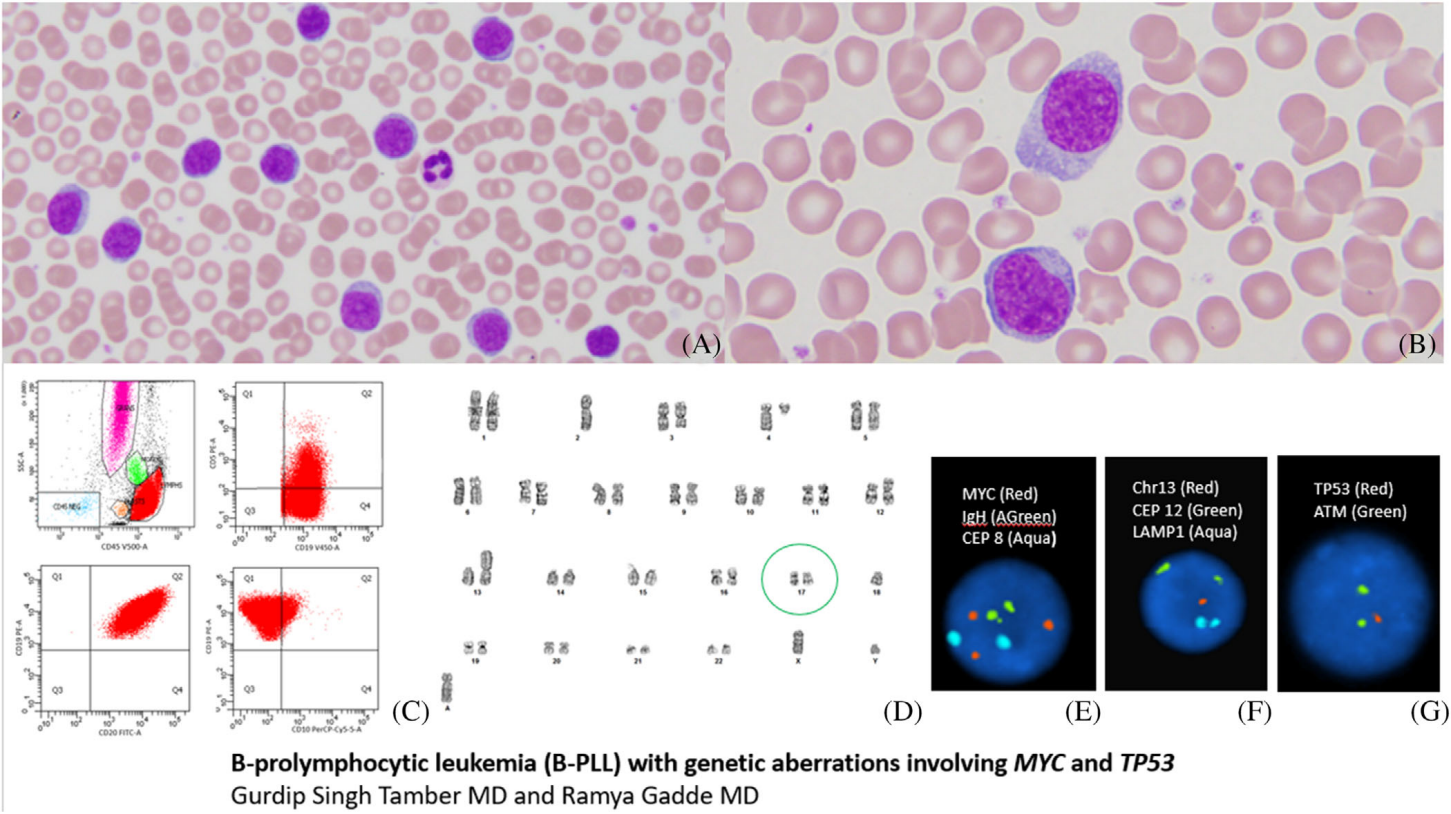


A 70‐year‐old man found to have leukocytosis of 44 × 10^9^/L with absolute lymphocytosis (9.37 × 10^9^/L) on routine peripheral blood (PB) testing with no B symptoms. CT chest/abdomen imaging showed splenomegaly and mild lymphadenopathy. Peripheral blood smear illustrated medium to large sized lymphocytes (90%, 8.5 × 10^9^/L) with scant agranular cytoplasm, ovoid nuclei with condensed chromatin and prominent nucleoli (panel A and B; hematoxylin and eosin stain, × 40 and × 100 objective respectively). Bone marrow (BM) examination identified an extensive infiltration of neoplastic prolymphocytes. Immunophenotyping of PB and BM showed kappa restricted B‐cell population positive for CD20, CD5 partial and weak and negative for CD10, CD23, CD200 (panel C, neoplastic B‐cells shown in red). Genetic work‐up revealed complex karyotype including del(17)(p12); FISH studies showed gain of *MYC*, 13q deletion and 17p deletion (panel D‐G). Molecular lymphoid NGS panel showed *TP53 p.Q167* (VAF 64%). The diagnosis B‐prolymphocytic leukemia (B‐PLL) was established. Although asymptomatic, patient was started on Ibrutinib.

B‐PLL is extremely rare with differential diagnosis including chronic lymphocytic leukemia with increased prolymphocytes, leukemic mantle cell lymphoma, and splenic marginal zone lymphoma. B‐PLL with *MYC* aberration and 17p *(TP53)* deletion have worst prognosis and should be treated aggressively. In these cases it is important to rule out Mantle cell lymphoma, FISH for t(11;14) should be performed which was negative in this case.

Increasing awareness of this entity may aid clinicians in a fast diagnostic workup, including immunophenotyping and genetic testing.

